# Endogenous fluctuations in cortical state selectively enhance different modes of sensory processing in human temporal lobe

**DOI:** 10.1038/s41467-023-41406-3

**Published:** 2023-09-11

**Authors:** Arun Parajuli, Diego Gutnisky, Nitin Tandon, Valentin Dragoi

**Affiliations:** 1 Department of Neurobiology and Anatomy, McGovern Medical School, University of Texas at Houston, Houston, TX USA; 2 Janelia Research Campus, Howard Hughes Medical Institute, Ashburn, VA USA; 3 Vivian L. Smith Department of Neurosurgery, University of Texas Medical School, Houston, TX USA; 4 Department of Electrical and Computer Engineering, Rice University, Houston, TX USA

**Keywords:** Sensory processing, Visual system

## Abstract

The degree of synchronized fluctuations in neocortical network activity can vary widely during alertness. One influential idea that has emerged over the past few decades is that perceptual decisions are more accurate when the state of population activity is desynchronized. This suggests that optimal task performance may occur during a particular cortical state – the desynchronized state. Here we show that, contrary to this view, cortical state can both facilitate and suppress perceptual performance in a task-dependent manner. We performed electrical recordings from surface-implanted grid electrodes in the temporal lobe while human subjects completed two perceptual tasks. We found that when local population activity is in a synchronized state, network and perceptual performance are enhanced in a detection task and impaired in a discrimination task, but these modulatory effects are reversed when population activity is desynchronized. These findings indicate that the brain has adapted to take advantage of endogenous fluctuations in the state of neural populations in temporal cortex to selectively enhance different modes of sensory processing during perception in a state-dependent manner.

## Abstract (web summary)

Task performance is known to vary with cortical state. Here, the authors use electrical recordings in human temporal lobe to show that the same cortical state can both facilitate and suppress performance in a task-dependent manner.

## Introduction

Cortical population activity exhibits endogenous fluctuations during alertness even in the absence of changes in behavioral context or sensory stimulation^1–3^. During active engagement in a behavioral task, fluctuations in population activity often range from the desynchronized to synchronized state^3,4^. These changes have been primarily reported in sensory^4–8^ and frontal cortical areas^9^ of various species and are typically linked to specific effects on behavioral performance. Indeed, local fluctuations in neural network synchrony were found to control the trial variability in population coding accuracy and behavioral performance when animals are engaged in a perceptual task. Specifically, previous work has shown that when ongoing local population activity is desynchronized, the correlated variability between neurons is reduced, and the accuracy of network and behavioral performance is enhanced^1,3,10^. This has led to the idea that the desynchronized cortical state may be the optimal state in which population coding accuracy and behavioral performance are most improved^3,11–14^. However, most previous studies have primarily focused on a specific mode of sensory processing, typically discrimination performance, and hence the generality of the hypothesized relationship between cortical state and behavior has remained unexplored.

Here, we performed electrical recordings from surface-implanted grid electrodes in the temporal lobe of humans to show that the same cortical state can both facilitate and suppress perceptual performance in a task-dependent manner. We found that when fluctuations in local population activity are synchronized, the encoding of incoming stimuli and perceptual performance are enhanced in a detection task and impaired in a discrimination task, but these task-dependent modulatory effects are reversed when population activity is desynchronized. This indicates that different modes of sensory processing are selectively facilitated in different states of population activity in temporal cortex during active perception.

## Results

**Fig. 1:**
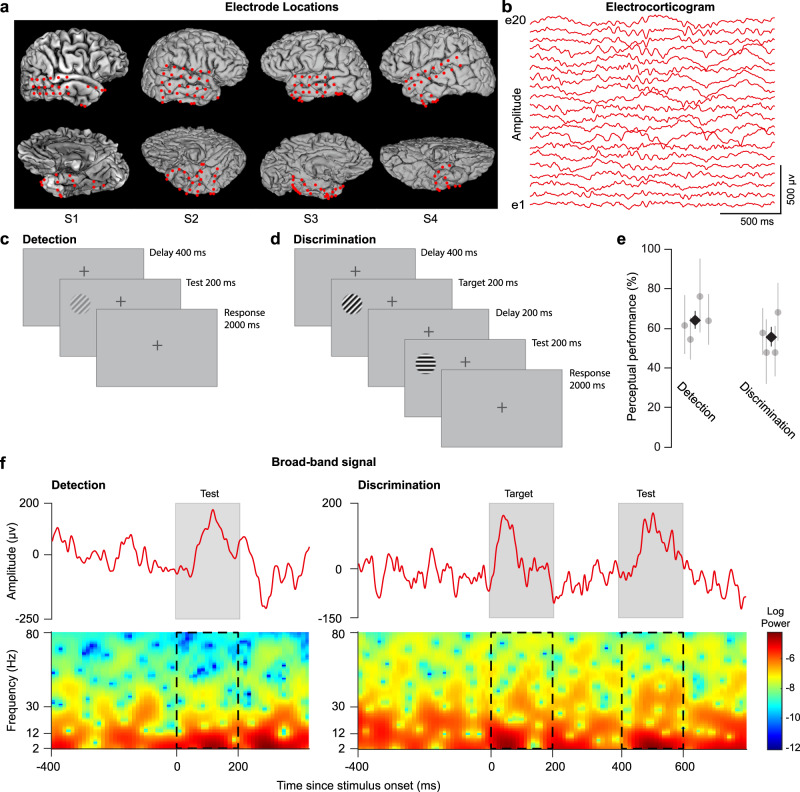
Experimental paradigm. **a** Locations of the electrodes in the temporal lobe for four subjects obtained by co-registering a post-operative CT scan with a pre-operative MRI structural image. **b** Example electrocorticogram (ECoG) recordings from 20 example electrodes from subject S1. **c**, **d** Perceptual tasks. Subjects performed contrast detection and orientation discrimination tasks while maintaining their fixation at the center of a grey background display, marked with a cross visible throughout the session. During the contrast detection task, a low-contrast (5% above detection threshold) sine-wave grating was displayed for 200 ms and subjects responded within the next 2000 ms by pressing a key if they were able to detect it. During the orientation discrimination task, a high-contrast (75%) sine-wave grating (target stimulus) of fixed orientation (45°) was presented for 200 ms, and after a delay of 200 ms, a slightly rotated (5% above discrimination threshold) grating (test stimulus) was presented for 200 ms. Subjects responded within 2000 ms by pressing a key if they judged the two stimuli to be different. **e** Average perceptual performance of four subjects during the detection (64.38 ± 4.5%, mean ± SEM, *n* = 4 subjects) and discrimination (55.83 ± 4.7%, mean ± SEM, *n* = 4 subjects) tasks (**f**) Top: broad-band ECoG signal during one example trial corresponding to the detection and discrimination tasks from one example electrode. Shaded regions represent stimulus presentation. Bottom: spectrogram of the signal from the top panel. Stimulus presentation is marked by dashed-line rectangles.

We recorded intracranial field potential (IFP) activity using multiple subdural electrodes from the temporal lobe of four human subjects (Fig. 1a, b, f) performing contrast detection and orientation discrimination tasks while they fixated at the center of a computer screen (see “Methods”). IFPs represent an indirect measure of cortical spiking activity, subthreshold activity, and electrical volume conduction from distant regions^10,15^. Prior to each experiment we measured the contrast detection and orientation discrimination thresholds for each subject (see “Methods”). The contrast detection task (Fig. 1c) consisted of a low-contrast (5% above threshold) sine-wave grating displayed for 200 ms, followed by a 2000-ms interval in which a behavioral response (button push) was recorded. The orientation discrimination task (Fig. 1d) consisted of two consecutive high-contrast (75%) sine-wave oriented gratings presented for 200 ms each and differing in orientation by an angle 5% above the discrimination threshold (the 2 stimuli were separated by a 200-ms delay, and subjects responded within 2000 ms to indicate whether the target and test gratings were different). To mark the start of each trial, a 20-ms sound was played 400 ms before the start of the first stimulus presented in each trial. No feedback of the outcome of the trial (correct/incorrect) was provided to the subjects. On average, perceptual performances for the detection and discrimination tasks were 64.38% ± 4.5% and 55.83% ± 4.7% (mean ± SEM), respectively (Fig. 1e), which allowed us to collect a relatively balanced number of correct and incorrect trials in each task. IFP signals from all the electrodes were analyzed, and only the electrodes that did not have spiking characteristics due to epilepsy (see “Methods”) were considered for further analysis (*n* = 97 electrodes).

### Power ratio (PR) as a measure of cortical state

**Fig. 2:**
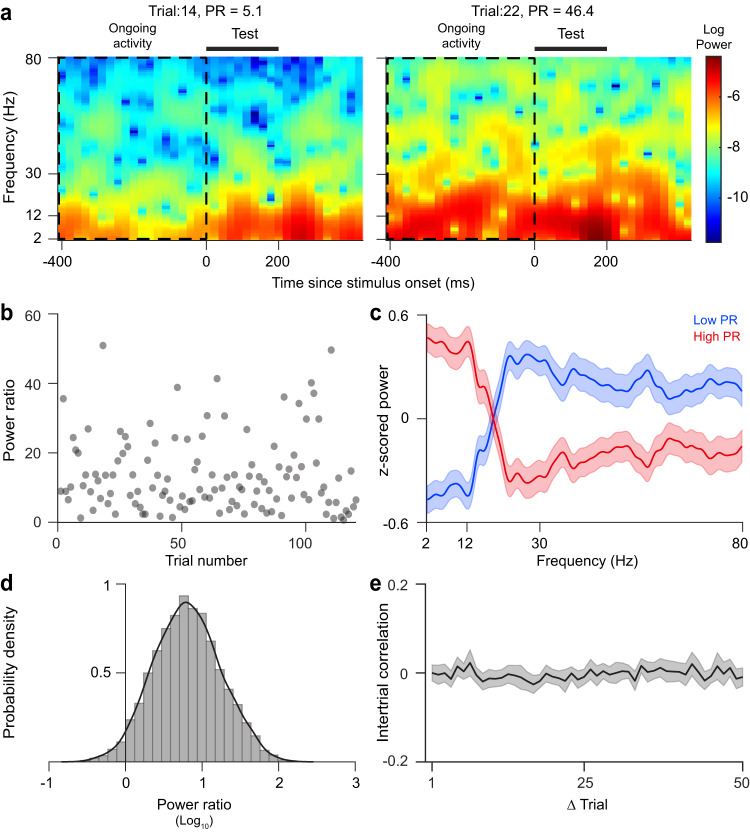
Measuring trial-by-trial fluctuations in cortical state during pre-stimulus interval. **a** Spectrograms from two example trials for one example electrode during the detection task reveal variability in the power of ongoing activity in different frequency bands (measured during the 400 ms window preceding the stimulus marked by the horizontal bar). **b** Trial-by-trial variability of the power ratio (PR) of pre-stimulus ongoing activity of an example electrode during 120 trials of the detection task. **c** Average z-scored spectrum of the ECoG signal during the 400-ms pre-stimulus period for the low and high PR groups of trials, using the same electrode presented as in (**b**). Trials were divided into low or high PR groups based on whether the PR value in a trial was below or above the median PR value of all the trials. **d** Probability distribution of pre-stimulus ongoing PR for all the electrodes considering both detection and discrimination tasks (10.62 ± 0.15, mean ± SEM.). **e** Average autocorrelation of pre-stimulus ongoing activity PR across trials for all the electrodes and sessions. Shaded area in all panels represents the standard error of mean.

Consistent with previous studies, the ‘synchronized’ cortical state was characterized by increased IFP power in the low frequency range and decreased power in the high frequency range^3,9,10,16–21^. Since the ratio of IFP low frequency power to high frequency power has been previously used to quantify the degree of synchronized fluctuations in a given trial^3,9,21^, we measured the power ratio (PR, see “Methods”) by calculating the ratio between the low (2.5–12 Hz) to high (12-80 Hz) frequency IFP power during the 400-ms pre-stimulus (ongoing) interval for each electrode. For most trials, IFP power in the low frequency band was higher than that in the high frequency band resulting in PR values predominantly greater than 1 (10.62 ± 0.15, mean ± SEM; Fig. 2d, see “Methods”). The distribution of PR values indicates that cortical state (defined as the mean PR across recording electrodes) is continuous rather than bimodal, in agreement with previous studies^10,14^. PR values followed a log-normal distribution, as evidenced by the normal distribution of logarithmic PR values presented in Fig. 2d; hence, we used log PR values in all statistical analyses performed here. Ongoing PR values for any electrode varied greatly from one trial to another (Fig. 2b). The autocorrelation of the ongoing PR values across trials did not reveal any significant temporal structure (Fig. 2e; *p* > 0.05, Wilcoxon rank-sum test, Bonferroni-corrected); the average autocorrelation of PR values across trials exhibited similar characteristics in both the detection and discrimination tasks (Fig. S3b). This implies that ongoing activity PR undergoes random fluctuations from trial to trial. Across trials, we found strong fluctuations in cortical population activity ranging from desynchronized to synchronized state, e.g., Fig. 2a which shows examples of desynchronized cortical activity in trial 14 and synchronized cortical activity in trial 22 for one example electrode recorded in the same session. There was no statistically significant difference in PR values between the detection and discrimination tasks (Fig. S3c, *p* > 0.05 for all subjects, two-sided Wilcoxon signed-rank test).

### Relationship between cortical state and perceptual decisions

**Fig. 3:**
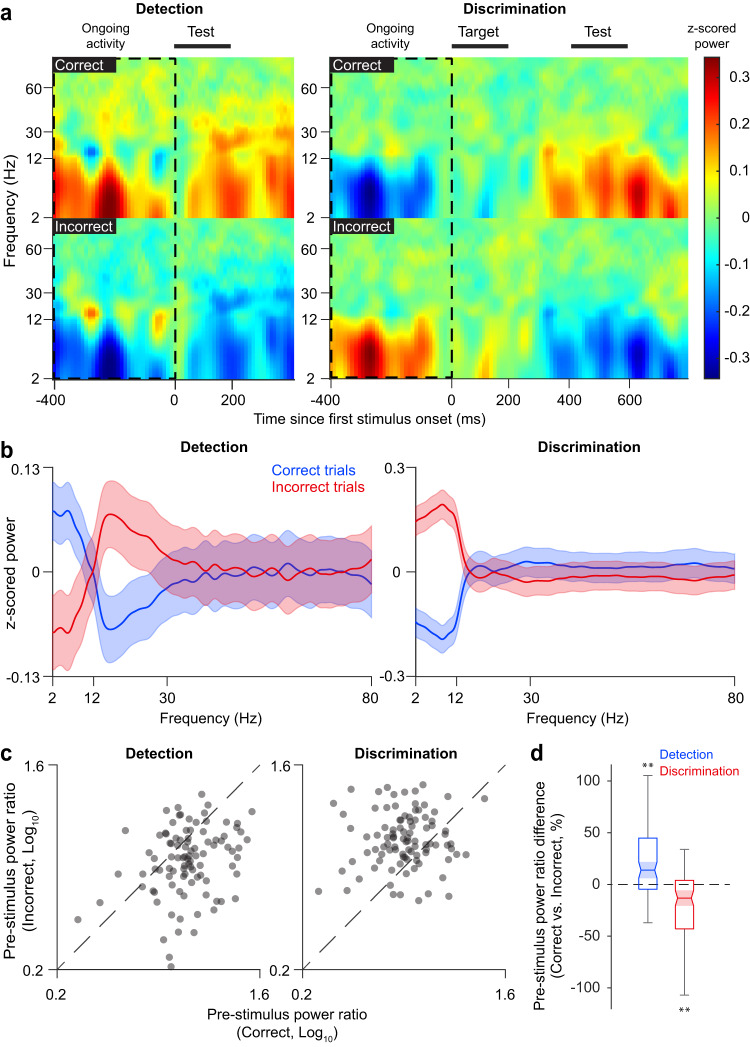
Task-dependent effects of cortical state on perceptual decisions. **a** Population average (*n* = 97) z-scored spectrograms for correct (top) and incorrect (bottom) trials during the detection (left) and discrimination (right) tasks showing the differences in ongoing activity between the two conditions in the 400-ms window preceding the stimulus marked by the dotted-line rectangles. **b** Population average (*n* = 97) z-scored spectra of ongoing activity in the 400-ms window preceding the stimulus for correct (blue) and incorrect (red) trials during the detection (left) and discrimination (right) tasks. Shaded areas represent the standard error of mean. **c** Comparison of pre-stimulus ongoing PR for correct and incorrect trials. Each dot represents one electrode (*n* = 97). In detection task, pre-stimulus ongoing PR is significantly higher for correct trials than for incorrect trials (*p* = 2.14e-07, two-sided Wilcoxon signed-rank test), whereas for the discrimination task, pre-stimulus ongoing PR is significantly lower for correct trials than for incorrect trials (*p* = 9.02e-09, two-sided Wilcoxon signed-rank test). The dotted lines represent the identity lines. **d** Population average (*n* = 97) of the percentage difference of ongoing PR between correct and incorrect trials for the detection (blue, 25.22 ± 4.48%, mean ± SEM; *p* = 2.03e-07, one-sided Wilcoxon signed-rank test for positive median) and discrimination (red, −21.25 ± 3.46%, mean ± SEM; *p* = 8.56e-08, one-sided Wilcoxon signed-rank test for negative median) tasks. Error bars represent standard error of mean. **p < 0.01. The box plot’s midline represents the median, the edges mark the quartiles, and the whiskers show the range. Notches indicate the 95% confidence interval of the median.

We further examined whether and how cortical state, measured by the PR value in the 400-ms period before stimulus presentation, influences the accuracy of perceptual responses. Thus, we divided trials into correct and incorrect response groups and then examined, for each electrode, the state of the ongoing pre-stimulus cortical activity (Fig. 3a, b, see also Figs. S1, S2) in each trial. Surprisingly, for the contrast detection task, correct trials were associated with significantly higher PR values than incorrect trials (difference = 25.22 ± 4.48%, mean ± SEM; *p* = 2.03e-07, one-sided Wilcoxon signed-rank test for positive median). However, this result was reversed for the discrimination task, whereby correct trials were associated with significantly lower PR values than incorrect trials (difference = −21.25 ± 3.46%, mean ± SEM; *p* = 8.56e-08, one-sided Wilcoxon signed-rank test for negative median, Fig. 3c, d, S6b). This task dependent difference in ongoing PR between correct and incorrect trials was found to be consistent across subjects (Figs. S4, S6a). Further examination of recording sites in six sub-regions of the temporal lobe (middle temporal gyrus, superior temporal gyrus, inferior temporal gyrus, temporal pole, fusiform gyrus, and parahippocampal gyrus) revealed that these task-dependent differences in ongoing PR values for correct and incorrect trials were present across the entire temporal lobe (Supplementary Table 1, Figs. S5, S6c). This indicates that the task-dependent effects of cortical state on perceptual performance are robust and consistent across temporal cortical subnetworks.

### Task-dependent influences of cortical state

**Fig. 4:**
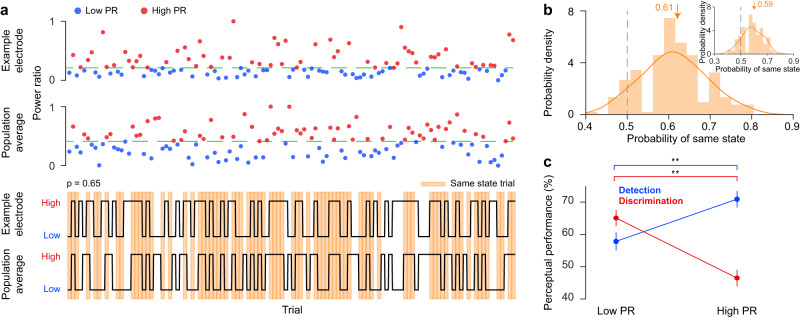
Same state probability and perceptual performance. **a** (Top) Normalized pre-stimulus ongoing power ratio (PR) for 120 detection task trials on one example electrode. The green dotted line represents the median. Trials below the median are in the low PR group (blue), and those above are in the high PR group (red). (Middle) The mean normalized PR of the remaining simultaneously recorded electrode population (excluding the example electrode at the top) for the same example session, divided into low PR (blue) and high PR (red) groups using the median value (green line). (Bottom) Black lines indicate the Low/High states for the example electrode (top) and the remaining population (bottom) for the 120 trials of the example session. High PR trials are above the session median, and low PR trials are below it. Orange shaded trials correspond to instances where the example electrode’s state matches that of the rest of the population. The example electrode had the same state as the remaining population in 65% of the trials. **b** Population distribution of same-state probabilities between individual temporal electrodes and the average of the rest of the population of the temporal electrodes. On average, individual electrodes shared the same state as the rest of the population for the majority of trials (0.61 ± 0.0054%, mean ± SEM, across all subjects). Inset: same-state probabilities between individual temporal electrodes and the average of all the electrodes from all the lobes (global state) of the brain. Arrows represent the mean. **c** Perceptual performance for detection (blue) and discrimination (red) tasks for low and high ongoing PR state trials. The high ongoing PR trials were associated with significantly better performance than the low ongoing PR trials for the detection task (low PR: 57.85 ± 2.81% vs. high PR: 70.92 ± 2.57%, mean ± SEM, *p* = 0.003, *n* = 97 electrodes, bootstrapping with 1,000,000 iterations). Conversely, for the discrimination task, the low ongoing PR trials were associated with significantly higher performance values than the high ongoing PR trials (low PR: 65.1 ± 2.48 vs. high PR: 46.5 ± 2.58, mean ± SEM, *p* < 1e-6, *n* = 97 electrodes, bootstrapping with 1,000,000 iterations). **p < 0.01.

We further investigated whether the pre-stimulus IFP power ratio (PR) at individual cortical sites is correlated with the state of the neural population, and hence examined the relationship between the trial-by-trial response state at a given recording site and the mean response of the rest of the electrodes recorded simultaneously in the same session. That is, for each recording site we computed the trial-by-trial ongoing activity PR, normalized between 0 and 1, and examined whether the PR of that electrode is correlated to the average PR of the rest of the population. In each trial, the cortical state at a given electrode location was classified as either “low” or “high” PR based on whether the PR value in that trial was below or above the median PR value at that electrode. By utilizing a similar methodology, we assessed the “low” or “high” PR state of the remaining cortical population during each of the trials (Fig. 4a). Based on the trial fluctuations in neural responses at individual recording sites, we calculated the probability that a given electrode is in the same pre-stimulus state as the population (Fig. 4a). For our group of recording sites, the mean probability of the ‘same’ cortical state was 0.61 ± 0.0054, mean ± SEM, across all subjects, i.e., significantly greater than chance level obtained by shuffling trials (chance level = 0.5, *p* = 1.54e-30, two-sided Wilcoxon rank-sum, Fig. 4b, S11), thus demonstrating that cortical state at individual recording sites is correlated with that of the entire temporal neural population that we recorded. To examine whether there is any difference in evoked response or spatial location between low and high state-correlated electrodes, we divided the recording sites into quartiles using their same-state probability values, and then examined their evoked response and location in the temporal lobe. We did not find statistically significant differences in evoked responses to test stimuli between the sites in the first quartile and those in the fourth quartile for both detection and discrimination tasks (Fig. S10a, b, Wilcoxon signed-rank test, *p* > 0.05). Furthermore, we found no differences in the probability of a low (first quartile) or high (fourth quartile) state-correlated electrode being located in any of the six-subregions of the temporal lobe (Fig. S10c, d, Kruskal-Wallis multiple comparison test by ranks).

To compare the state-correlations among temporal sites to that between temporal sites and the whole brain, we measured global brain state by pooling all the electrodes exhibiting non-epileptic activity (not only those in the temporal lobe) that were surgically implanted (see “Methods”). This allowed us to measure the correlation between low and high PR states of temporal lobe electrodes and global brain state (i.e., the average of the ‘state’ of recording sites from all brain areas), and hence calculate the same-state probability between each temporal lobe electrode and the average of all other electrodes. Our analysis in Fig. 4b (inset) shows that the same-state probability of temporal lobe electrodes to global brain state was 0.59 ± 0.0055 (mean ± SEM), which is statistically significant, but lower than the same-state probability of temporal lobe electrodes to the rest of the electrodes within the same lobe (Fig. 4b, 0.61 ± 0.0053, mean ± SEM; *p* < 1.4e-410, Wilcoxon signed-rank test). This analysis suggests the presence of significant correlation between global brain state and local fluctuations of neural activity in temporal cortex.

We further classified each trial into ‘low’ or ‘high’ PR depending on whether the average PR during a trial was below or above the median of the PR values across trials. Since the PR values of individual electrodes are significantly correlated with the mean PR of the neural population (Fig. 4a, b), we reasoned that the mean population PR represents a viable measure of the cortical state of the entire population. We then looked at the perceptual performance of the subjects for each task separately for the low and high PR trial groups. Consistent with the results in Fig. 3, detection performance was significantly elevated in the high PR trials (low PR: 57.85 ± 2.81% vs. high PR: 70.92 ± 2.57%, mean ± SEM, Fig. 4c, S8), whereas discrimination performance was significantly elevated in the low PR trials (low PR: 65.1 ± 2.48 vs. high PR: 46.5 ± 2.58, mean ± SEM, Fig. 4c, S8). Next, we divided the range of PR values into three equal-size bins and examined the change in perceptual performance. This analysis revealed similar effects of PR on perceptual performance as with two PR groups, i.e., during the detection task, perceptual performance increased with increasing PR values, whereas during the discrimination task, perceptual performance decreased with increasing PR values (Fig. S7). We further examined the relationship between the pre-stimulus PR values and reaction times during correct trials for the two tasks. As expected, we found a significant negative correlation between reaction time and PR values (r = −0.042, *p* = 0.006, one-sided Wilcoxon signed-rank test, Fig. S9) in the detection task, indicating that subjects responded more promptly when PR values were higher (in the synchronized state). In contrast, we found a significant positive correlation between reaction time and PR values (r = 0.11, *p* = 2.4e-5, one-sided Wilcoxon signed-rank test, Fig. S9) in the discrimination task, indicating that subjects responded more promptly when the PR values were lower (in the desynchronized state). This indicates that, for each subject, cortical state modulates perceptual performance in a task-dependent manner. That is, the synchronized cortical state, currently believed to be detrimental for perceptual decisions, is in fact beneficial for sensory detection but detrimental for sensory discrimination.

### Relationship between cortical state and evoked neural activity

**Fig. 5:**
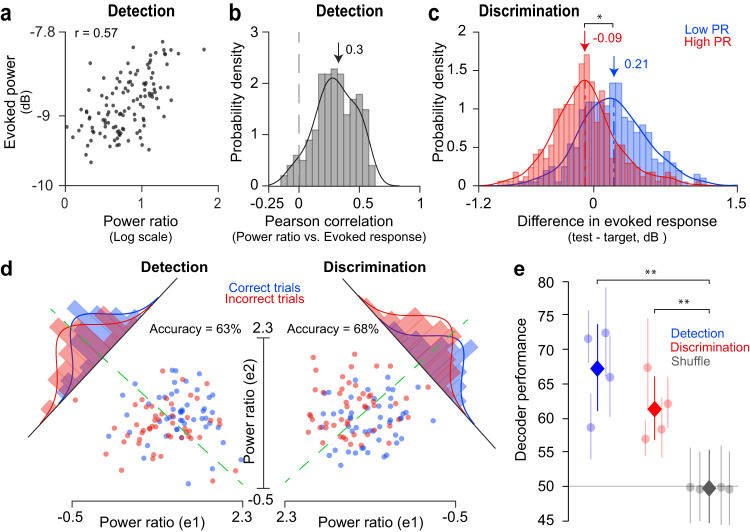
Cortical state predicts behavioral outcome. **a** Relationship between ongoing PR and stimulus evoked response for one example electrode during the detection task. Stimulus evoked response is positively correlated with pre-stimulus ongoing PR (Pearson’s correlation = 0.57, *p* < 1.0e-9). **b** The Pearson’s correlation probability density function for PR and evoked response across all subjects and electrodes in both tasks (0.3 ± 0.01, mean ± SEM; *p* = 6.51e-33, one sided Wilcoxon signed-rank test for positive median). **c** Population distribution of the difference in evoked responses to the target and test stimuli (test – target) for low (blue) and high (red) PR trials. Median PR value during a session was used to divide trials into low and high PR groups. The difference was significantly larger for the low PR trials than for the high PR trials (0.21 ± 0.02 dB vs. −0.09 ± 0.02 dB, mean ± SEM; *p* = 0.02, tow-sided Wilcoxon signed-rank test) (**d**) Fisher-Linear Discriminant Analysis (F-LDA) for the same pair of example electrodes during detection and discrimination tasks. Each dot represents the ongoing PR during a correct (blue) or incorrect (red) trial. The green dotted lines represent the decision boundaries of the trained F-LDAs for the two tasks. Histograms are generated by projecting each PR value onto the F-LDA axis so that the separability between correct and incorrect histograms is maximized. The curves around histograms represent one-dimensional Gaussian fits for the respective histograms. **e** Population average of the F-LDA performance during the detection (blue) and discrimination (red) tasks. Performance is significantly above the chance level (gray) for both detection (67.38 ± 6.39, mean ± SEM; *p* = 0.003, bootstrapping with 10,000 iterations) and discrimination (61.42 ± 4.7, mean ± SEM; *p* = 0.007, bootstrapping with 10,000 iterations) tasks. The F-LDA was trained to predict whether a subject was going to be correct or incorrect in a trial based on the pre-stimulus ongoing PR values. **p < 0.01.

We further examined the impact of cortical state on the evoked neural population response in each task. First, we found a significant positive correlation between stimulus-evoked responses – as quantified by the voltage-squared power of the broad-band ECoG signal during the first stimulus – and the PR of the pre-stimulus neural activity (400-ms before stimulus presentation, Fig. 5a, b; r = 0.3 ± 0.01, mean ± SEM; Pearson correlation; *p* = 6.51e-33, one-sided Wilcoxon signed-rank test for positive median). This is consistent with signal detection theory^22^ postulating that the visibility of a stimulus near the physical limit of detectability in any single trial depends on whether the neural response evoked by the stimulus crosses a threshold. This indicates that stimuli evoking stronger responses would be detected, but those evoking smaller responses would not^22,23^. Overall, this is consistent with our findings that correct responses in the contrast detection task are associated with the synchronized cortical state.

Second, we reasoned that discrimination performance may be related to the difference between the neural population responses elicited by the target and test stimuli. That is, improved discrimination performance during the desynchronized cortical state (low power ratio trials) might occur because of a larger difference in the two evoked responses in this group of trials. To test this hypothesis, we divided trials in each session into low and high PR groups (based on the median PR value extracted from the pre-stimulus response), and then calculated the difference in evoked responses to the target and test stimuli (test – target) for those two groups of trials (Fig. 5c). Confirming our hypothesis, and consistent with previous work in rodents^8,13,24,25^ and monkeys^3^, the evoked response difference was significantly higher for the desynchronized trials than for synchronized trials (0.21 ± 0.02 dB vs. −0.09 ± 0.02 dB, mean ± SEM; *p* = 0.02, two-sided Wilcoxon signed-rank test), thus indicating that the improvement in discrimination performance is associated with the desynchronized state.

Finally, we examined whether cortical state measured during the 400-ms pre-stimulus window can be used to predict perceptual performance on a trial basis regardless of the stimulus evoked response. Thus, a Fisher-Linear Discriminant Analysis decoder (F-LDA, see “Methods”) was trained to predict whether a trial will be correct or not in a given task based on the pre-stimulus ongoing PR values. Confirming our prediction, we found that, for each subject, decoder performance was significantly above chance level (0.5) for both detection and discrimination tasks (Fig. 5d, e; detection: 67.38 ± 6.39, mean ± SEM; discrimination: 61.42 ± 4.7, mean ± SEM). This provides further support for our hypothesis that cortical state modulates perceptual performance in a task-dependent manner.

## Discussion

Previous studies examining the impact of cortical state on behavioral performance have revealed that synchronous fluctuations in population activity decrease the accuracy of neural network computations and perceptual accuracy even when the animal is seemingly alert and actively engaged in the task^3,10–13,26–28^. However, these influences have been primarily revealed when animals performed a single task, most often discrimination, but whether or not there are optimal cortical states for particular types of behavior has remained unknown. We performed novel experiments in human temporal cortex to demonstrate that different modes of sensory processing are selectively facilitated in different states of population activity during active perception. When local population activity is synchronized, network and behavioral performance are enhanced in a detection task and impaired in a discrimination task, but these modulatory effects are reversed when population activity is desynchronized. Notably, the endogenous fluctuations in temporal cortex recorded during the perceptual task were significantly correlated with global brain state.

Recent work in mouse and monkey sensory cortex has explored the impact of cortical state on neuronal responses and behavior^1,25^. However, there are important differences between our study and earlier investigations. Specifically, besides the fact that our experiments were performed in humans, our definition of cortical state is restricted to the local population activity monitored while the subject performed the task. In contrast, previous investigations measured global fluctuations in behavioral state, such as arousal, and their impact of neuronal responses. For instance, it was reported that rapid variations in locomotion and arousal (as measured by pupil diameter) control sensory evoked responses and spontaneous activity of individual neurons^1,29^, and that noise correlations were lower during locomotion compared with quiescence, while evoked responses were stronger^1,29^. Global state fluctuations, such as arousal, were also found to modulate the membrane potentials of auditory cortical neurons in mice trained in a tone-in-noise detection task^30^. Importantly, arousal level was found to modulate behavioral performance by enhancing sensory-evoked cortical responses and reducing background synaptic activity. However, previous studies did not investigate whether fluctuations in population synchrony influence the information encoded by ensembles of neurons and behavioral performance in a task-dependent manner.

Although the type of task dependent influences of cortical state on perceptual performance reported here have not been described before, state-dependent effects on sensory processing have been shown in previous animal studies. For instance, whisker deflection stimuli presented in isolation were found to evoke larger responses in the synchronized relative to desynchronized state in somatosensory cortex^6,25,31–34^. However, the ability of somatosensory neurons to accurately represent complex whisker deflections was improved in the desynchronized state^13,24,25^. Furthermore, our finding that the difference in evoked responses elicited by successive stimuli presented during orientation discrimination is increased in the desynchronized cortical state is consistent with previous results in auditory cortex whereby the impact of successive stimuli was assessed in different states of engagement, passive or active^8,35^. This suggests that rapidly repeated stimuli are “filtered out” in synchronized states, but efficiently processed in the desynchronized state^4^. Finally, our results are consistent with previous results in mouse and monkey visual cortex reporting that the desynchronized cortical state is optimally suited for visual discrimination and enhanced accuracy of neural populations^2,3,36^. Nonetheless, none of these previous studies have shown that cortical state can be both facilitatory and suppressive depending on the nature of the behavioral task.

Why would it be advantageous for humans to have better detection performance during the synchronized cortical state and better discrimination performance in the desynchronized state? We reasoned that during natural behavior, the fine details of environmental stimuli may be of little relevance to a resting individual when the brain is in a more synchronized state^3,9^. Therefore, during rest or passive wakefulness ongoing stimuli will not be lost since endogenously generated neural patterns characterizing the synchronized state facilitate stimulus detection, and sudden unexpected sounds or touch stimuli will trigger an immediate behavioral response^32,37^. On the other hand, during the desynchronized brain state, when individuals are likely to be active, the ability to identify small changes in the environment by discriminating ongoing stimuli will be beneficial for active behavior. This further suggests that the brain has adapted to take advantage of endogenous fluctuations in cortical state to differentially enhance different modes of sensory processing during perception in a state-dependent manner.

Could our state-dependent results generalize to other cortical areas or they are specific to temporal cortex? For instance, previous work has suggested that a reduction in noise correlations in visual cortex^38^ and superior colliculus^37^, which may be associated with the desynchronized cortical state, is beneficial for visual detection. Unfortunately, in our study we were unable to examine multiple brain areas since the placement of recording electrodes was clinically motivated, and hence we could not rely on a sufficiently large number of recording sites in other areas (outside temporal lobe) in all patients to test the generality of our state-dependent effects. Regardless, the fact that neural populations in temporal cortex, and not other areas, take advantage of fluctuations in cortical state to differentially enhance different modes of sensory processing during perception might reflect the fact that neurons in human temporal cortex are tuned to perceptual processes based on a fine-grained analysis of the identity of visual stimuli. Therefore, temporal cortical neurons may use the desynchronized state, generally associated with high arousal and attention, to perform more demanding visual tasks, such as fine stimulus discriminations.

One potential concern is that our study was performed in epileptic human patients. Epilepsy is a disease marked by impaired cognitive and memory performance often as a result of increased synchronized activity of large numbers of neurons. However, there are at least two reasons why the neurological condition of the subjects is unlikely to be a confounding issue in our study. First, we systematically removed electrodes showing ictal and interictal discharge from our analysis, as evaluated by our clinical team, and carefully inspected all analyzed trials for artifacts related to epilepsy (see Methods). Additionally, if disease-related low-frequency synchronization was indeed impairing brain function and ictal discharge alone accounted for our results, we would anticipate an abnormal increase in low-frequency activity, which would lead to high PR values across all trials. However, our findings do not support this hypothesis. Furthermore, all experiments were conducted in the morning when subjects did not exhibit any signs of epilepsy and to ensure that subjects were eligible to participate in our psychophysics experiments, they were tested by our clinical team before each experiment. Considering all these facts, we can be confident that the neurological conditions of the subjects are not confounding the results of our study.

Taken together, our results demonstrate that the structure of variability in activity of local cortical populations in the temporal lobe is not noise but rather influences sensory processing in a task-dependent manner. However, to elucidate exactly how these cortical state effects are manifested to influence perception and cognition, future studies are needed to causally manipulate the state of the cortex and measure its impact on the trial-by-trial population code and behavioral responses. Importantly, it remains to be seen whether the trial fluctuations in temporal cortical populations are coordinated across visual cortical areas, and whether the multi-area spatiotemporal pattern of population activity is relevant for behavior. Future research will also elucidate whether the task-dependent relationship between the fluctuations in the state of neural populations and perceptual performance reported here is a component of a more general coding strategy across the visual system, and possibly other sensory pathways.

## Methods

### Subjects

Intracranial Field Potential (IFP) recordings were made using Electrocorticography (ECoG) from 4 patients (3 males, 1 female; 37.75 ± 7.95 years old, mean ± SEM; 2 right-handed, 2 left-handed; 3 right implant, 1 left implant) with medically intractable epilepsy. The patients had subdural electrodes implanted on the cortical surface of the brain to aid in the localization of seizure foci, with the aim of identifying potential targets for surgical resection. The electrodes had the following spatial localization: S1: 23 temporal lobe, 55 non-temporal lobe (16 frontal, 15 parietal, 24 occipital); S2: 34 temporal lobe, 45 non-temporal lobe (30 frontal, 14 parietal, 1 occipital); S3: 22 temporal lobe, 24 non-temporal lobe (21 frontal, 2 parietal, 1 occipital); S4: 18 temporal lobe, 79 non-temporal lobe (62 frontal, 17 parietal, 0 occipital). All subjects were admitted into Memorial Hermann Hospital, Houston and all the procedures used in the experiment were approved by the Institutional Review Board of the hospital. Each patient had given informed consent about the experiments approved by the University of Texas, Medical School at Houston committee for the protection of human subjects.

### Electrical recordings

Electrophysiological methods and electrode localization were similar to those described previously^39,40^. In brief, subdural circular platinum-iridium electrodes with a top hat design (4.5-mm overall diameter, 3-mm cortical contact, 10-mm interelectrode distance) were implanted and placed solely on the basis of clinical considerations using standard techniques^41^. Electrode localization was verified by co-registering a post-operative CT imaging with a pre-operative MRI structural image. Lobar and gyral labels were assigned by an expert in human neuroanatomy (N.T.). ECoG signals were sampled at 1000 Hz using Nihon Kohden NeuroFax (Japan) with a recording bandwidth from 0.15 to 300 Hz. Signals were referenced to a common average consisting of all non-ictal electrodes over lateral frontal and lateral temporal areas to minimize the effect of the referencing scheme on synchronization measures^42^. Recordings were then imported into MATLAB for post-processing. All the data used in the analysis originated from recordings free from seizure events.

### Anatomical imaging

Anatomical imaging data was acquired with a 3 T whole-body MR scanner (Philips Medical Systems, Bothell WA) equipped with a 16-channel SENSE head coil prior to surgery. A magnetization-prepared 180^o^ radio-frequency pulses and rapid gradient-echo (MP-RAGE) sequence with 1 mm thick sagittal slices and an in-plane resolution of 0.938 × 0.938 mm and functional MRI volumes (thirty-three axial slices, 3 mm slice thickness, 2.75 in-plane resolution, 30 ms TE, 2015 ms TR, 90° flip angle) were collected. For each subject, a 3-dimensional reconstruction of the pial surface was generated using FreeSurfer v4.5^43^. Subdural electrodes (SDEs) were localized on the surface using CT scans taken after implantation and intra-operative photographs at the time of grid placement and resection^44^. For representation in a common coordinate space, the SDEs were displayed in the MNI-N27 surface using a 12-parameter affine transformation.

### Epileptic spiking detection

For each task, we computed the *mean* and standard deviation (*std*) of the absolute values of the raw ECoG signal (by concatenating all the trials) in order to compute a threshold *TH* = *mean* + 3*std. Subsequently, for each trial, we computed the time interval for which the absolute raw signal was greater than *TH*. “Spiking trials”, which were defined as those trials for which the absolute raw signal was >*TH* for at least 5% of the time, were removed from the analysis. The entire electrode was excluded from further analysis if the number of trials with ‘normal (non-epileptic) activity was <50 in either of the detection or discrimination task.

### Behavioral tasks

Experiments consisted of *Contrast detection* and *Orientation discrimination* tasks performed in the same session for each subject (their order was randomly interleaved across subjects). In each task, subjects fixated at the center of the screen on a 0.2 deg dot while visual stimuli were presented contralaterally with respect to the hemisphere where electrodes were implanted (around 5 deg eccentricity with respect to the fixation point). Subjects sat comfortably in front of a computer screen about 60 cm away and each one of them had a chin rest to facilitate performance. Eye position was continuously monitored using an infrared eye tracker for humans (Iscan) operating at 60 Hz. A trial was aborted if fixation was broken before the end of the trial, or fixation instability exceeded 0.5 deg. We did not find any statistically significant differences in eye position and velocity between correct and incorrect trials in any subject (*P* > 0.1, Wilcoxon sign rank test). Before each recording session, subjects participated in two separate staircase experiments to determine the threshold contrast value for the detection task and the threshold orientation difference for the discrimination task. The threshold values in each session were used to generate the actual stimuli presented in the experimental sessions. For both tasks, a 20-ms sound was played 400 ms before the start of the first visual stimulus that was presented. *Contrast detection* task consisted of detecting a low contrast (5% above detection threshold) 4-deg sine-wave grating presented for a period of 200 ms. The *Orientation discrimination* task required subjects to discriminate between a high-contrast (75%) sine-wave grating (target stimulus) and the same grating, slightly rotated (5% above discrimination threshold), test stimulus flashed after a 200-ms delay (stimulus size was identical in both experiments and across subjects). Both stimuli were displayed for 200 ms and the target had a fixed orientation of 45° (Fig. 1c, d). Subjects responded within 2000 ms time window after the offset of the test stimulus by pressing a key if they perceived a stimulus (contrast detection) or a change in orientation (orientation discrimination). The number of trials in each task varied between 60 and 120 trials.

### Data analysis

All the analysis of the data was performed using custom software developed using MATLAB (MathWorks, Natick, MA), Python programming language (Python Software Foundation, https://www.python.org/) and SciPy^45,46^.

### Electrode localization and selection

Electrodes were localized to sub-regions of the brain by co-registering a post-operative CT imaging with a pre-operative MRI structural image. We focused our analysis on IFP recordings from temporal lobe electrodes from four subjects as temporal lobe had the best electrode coverage in all of the subjects. The IFP signals from all the electrodes were analyzed and only the electrodes that did not have spiking characteristics due to medical conditions were considered for further analysis (total 97 electrodes).

### Data referencing and filtering

In each recording session, we used the average of the IFP signals from all nonictal electrodes as the reference value for each individual electrode. The unfiltered IFP recording was first treated to remove the line-noise at 60 Hz by filtering with Chebyshev-Type-II filter with 60db attenuation in the frequency range 60 ± 0.6 Hz. The IFP signal was further filtered into low (2.5–12 Hz) and high (12–80 Hz) frequency bands for subsequent analysis. We applied a Chebyshev-Type-II bandpass filter, setting the cutoff frequencies at f1-0.3 Hz and f2 + 0.3 Hz. Here, f1 and f2 denote the lowest and highest frequencies of the passband, respectively. Additionally, we ensured a stopband attenuation of 60 db. To eliminate any phase delay introduced by the filter, we applied a double filtering process. First, we filtered the signal and then reversed it. Next, we applied a second filter to the reversed signal and again reversed it. By doing so, we ensured that any phase shift introduced by the filter was effectively nullified. To account for potential variations in recording characteristics across different sessions, we performed z-transform normalization on the IFP signal for each channel. This normalization step was carried out whenever we pooled the channels to compute population statistics. By doing so, we reduced the likelihood of any few channels disproportionately affecting the overall population statistics, thus ensuring a more accurate and reliable analysis.

### z-scored spectrum and spectrogram

To compare the power of pre-stimulus IFP signal at each frequency between correct and incorrect conditions, power spectrum of the signal was computed at each electrode for all trials using Chronux analysis software^47^. The power at each frequency was then z-scored for each electrode, taking all the trials into account, so that the mean of the powers for all the trials at each frequency was made equal to zero. The trials were then separated into correct and incorrect groups, and the mean value for each group was calculated to obtain the z-scored spectrum for the correct and incorrect conditions. Due to the z-scoring process, which equalized the mean to zero, the z-scored spectra for correct and incorrect conditions added up to zero at each frequency point resulting in a mirror symmetry around zero. The z-scored spectrogram was computed using the same method for each time point i.e., by z-scoring along the trial dimension for each time and frequency point.

### Power ratio (PR)

Power ratio (PR) of IFP signal was defined as the ratio of power in the lower frequency band (2.5–12 Hz) to that in the higher frequency band (12–80 Hz). To estimate the IFP power within a specific frequency band of interest, we first applied a corresponding band-pass filter to the signal and then computed the average voltage-squared power of the resulting band-passed signal. PR of the ongoing IFP in the 400 ms time window preceding the first stimulus was computed to determine the cortical state in each trial.

### Fisher-linear discriminant analysis (LDA)

Equal numbers of correct and incorrect trials were randomly selected from each recording session to train a Fisher-Linear Discriminant Analysis (F-LDA)^48,49^ with five-fold cross-validation for predicting whether the subject was going to be correct or incorrect in a given trial based on the ongoing pre-stimulus PR of IFP signals from the temporal electrodes. This procedure was repeated 10,000 times to get an estimate of the mean and standard deviation of the performance of the F-LDA. The number of times the F-LDA’s performance was below 50% divided by the total number of iterations (10,000) gave the *p*-value for statistical significance testing.

### Reporting summary

Further information on research design is available in the Nature Portfolio Reporting Summary linked to this article.

### Supplementary information

Supplementary Information

Reporting Summary

### Source data

Source Data

## Data Availability

Source data used to generate the main and supplementary figures are provided as a Source Data file and can be accessed here https://zenodo.org/record/8241829. The raw datasets generated as part of this research are not publicly available due to containing information that does not comply with HIPAA regulations, and the human participants from whom the data were collected have not consented to their public release. However, they are available from the corresponding author upon request. Source data are provided with this paper.

## Code availability

Manual codes used for data analysis can be accessed here: https://zenodo.org/record/8190383.

## References

[1] Vinck M, Batista-Brito R, Knoblich U, Cardin JA (2015). Arousal and locomotion make distinct contributions to cortical activity patterns and visual encoding. Neuron.

[2] Gutnisky DA, Beaman C, Lew SE, Dragoi V (2017). Cortical response states for enhanced sensory discrimination. Elife.

[3] Beaman CB, Eagleman SL, Dragoi V (2017). Sensory coding accuracy and perceptual performance are improved during the desynchronized cortical state. Nat. Commun..

[4] Harris KD, Thiele A (2011). Cortical state and attention. Nat. Rev. Neurosci..

[5] Worgotter F (1998). State-dependent receptive-field restructuring in the visual cortex. Nature.

[6] Fanselow EE, Nicolelis MAL (1999). Behavioral modulation of tactile responses in the rat somatosensory system. J. Neurosci..

[7] Castro-Alamancos MA, Oldford E (2002). Cortical sensory suppression during arousal is due to the activity-dependent depression of thalamocortical synapses. J. Physiol..

[8] Otazu GH, Tai L-H, Yang Y, Zador AM, Author NN (2009). Engaging in an auditory task suppresses responses in auditory cortex HHS Public Access Author manuscript. Nat. Neurosci..

[9] Milton R, Shahidi N, Dragoi V (2020). Dynamic states of population activity in prefrontal cortical networks of freely-moving macaque. Nat. Commun..

[10] Jacobs EAK, Steinmetz NA, Peters AJ, Carandini M, Harris KD (2020). Cortical state fluctuations during sensory decision making. Curr. Biol..

[11] Goard M, Dan Y (2009). Basal forebrain activation enhances cortical coding of natural scenes. Nat. Neurosci..

[12] Marguet SL, Harris KD (2011). State-dependent representation of amplitude-modulated noise stimuli in rat auditory cortex. J. Neurosci..

[13] Zagha E, Casale AE, Sachdev RNS, McGinley MJ, McCormick DA (2013). Motor cortex feedback influences sensory processing by modulating network state. Neuron.

[14] Zagha E, McCormick DA (2014). Neural control of brain state. Curr. Opin. Neurobiol..

[15] Buzsáki G, Anastassiou CA, Koch C (2012). The origin of extracellular fields and currents-EEG, ECoG, LFP and spikes. Nat. Rev. Neurosci..

[16] Noda H, Manohar S, Ross Adey W (1969). Correlated firing of hippocampal neuron pairs in sleep and wakefulness. Exp. Neurol..

[17] Noda H, Ross Adey W (1970). Changes in neuronal activity in association cortex of the cat in relation to sleep and wakefulness. Brain Res..

[18] Coenen A, Zajachkivsky O, Bilski R (1998). In the footsteps of Beck: the desynchronization of the electroencephalogram. Electroencephalogr. Clin. Neurophysiol..

[19] Niell CM, Stryker MP (2010). Modulation of visual responses by behavioural state in mouse visual cortex. Neuron.

[20] Li CYT, Poo MM, Dan Y (2009). Burst spiking of a single cortical neuron modifies global brain state. Science (80-.).

[21] Poulet JFA, Crochet S (2019). The cortical states of wakefulness. Front. Syst. Neurosci..

[22] Green DM, Swets JA (1966). Signal detection theory and psychophysics. New York Wiley.

[23] Sederberg AJ, Pala A, Zheng HJV, He BJ, Stanley GB (2019). State-aware detection of sensory stimuli in the cortex of the awake mouse. PLoS Comput. Biol..

[24] Hasenstaub A, Sachdev RNS, McCormick DA (2007). State changes rapidly modulate cortical neuronal responsiveness. J. Neurosci..

[25] McGinley MJ (2015). Waking state: rapid variations modulate neural and behavioral responses. Neuron.

[26] McCormick DA, Nestvogel DB, He BJ (2020). Neuromodulation of brain state and behavior. Annu. Rev. Neurosci..

[27] Nandy, A., Nassi, J. J., Jadi, M. P. & Reynolds, J. Optogenetically induced low-frequency correlations impair perception. *Elife* **8**, e35123 (2019). 10.7554/eLife.35123 PMC6391072 30794156

[28] Neske GT, Nestvogel D, Steffan PJ, McCormick DA (2019). Distinct waking states for strong evoked responses in primary visual cortex and optimal visual detection performance. J. Neurosci..

[29] Erisken S (2014). Effects of locomotion extend throughout the mouse early visual system. Curr. Biol..

[30] McGinley MJ, David SV, McCormick DA (2015). Cortical membrane potential signature of optimal states for sensory signal detection. Neuron.

[31] Crochet S, Petersen CCH (2006). Correlating whisker behavior with membrane potential in barrel cortex of awake mice. Nat. Neurosci..

[32] Ferezou I (2007). Spatiotemporal dynamics of cortical sensorimotor integration in behaving mice. Neuron.

[33] Hentschke H, Haiss F, Schwarz C (2006). Central signals rapidly switch tactile processing in rat barrel cortex during whisker movements. Cereb. Cortex.

[34] Krupa DJ, Wiest MC, Shuler MG, Laubach M, Nicolelis MAL (2004). Layer-specific somatosensory cortical activation during active tactile discrimination. Science (80-.).

[35] Castro-Alamancos MA (2004). Absence of rapid sensory adaptation in neocortex during information processing states. Neuron.

[36] Gutnisky DA, Beaman CB, Lew SE, Dragoi V (2017). Spontaneous fluctuations in visual cortical responses influence population coding accuracy. Cereb. Cortex (New York, NY).

[37] Cohen JD, Castro-Alamancos MA (2010). Behavioral state dependency of neural activity and sensory (whisker) responses in superior colliculus. J. Neurophysiol..

[38] Andrei AR, Pojoga S, Janz R, Dragoi V (2019). Integration of cortical population signals for visual perception. Nat. Commun..

[39] Conner CR, Ellmore TM, Pieters TA, di Sano MA, Tandon N (2011). Variability of the relationship between electrophysiology and BOLD-fMRI across cortical regions in humans. J. Neurosci..

[40] Chelaru MI (2016). Reactivation of visual-evoked activity in human cortical networks. J. Neurophysiol..

[41] Tandon, N. Cortical mapping by electrical stimulation of subdural electrodes: language areas. in *Textbook of Epilepsy Surgery* (ed. Luders, H. O.) 1001–1015 (CRC Press, 2008).

[42] Nunez, P. L. & Srinivasan, R. *Electric Fields of the Brain*. (Oxford University Press, 2006). 10.1093/acprof:oso/9780195050387.001.0001.

[43] Dale AM, Fischl B, Sereno MI (1999). Cortical surface-based analysis. Neuroimage.

[44] Pieters TA, Conner CR, Tandon N (2013). Recursive grid partitioning on a cortical surface model: an optimized technique for the localization of implanted subdural electrodes. J. Neurosurg..

[45] Virtanen P (2020). SciPy 1.0: fundamental algorithms for scientific computing in Python. Nat. Methods.

[46] Hunter JD (2007). Matplotlib: A 2D graphics environment. Comput. Sci. Eng..

[47] Mitra, P. and Bokil, H. Chronux Analysis Software, Chronux Home. Available at: http://chronux.org/ (Accessed: 15 March 2023).

[48] Tharwat A, Gaber T, Ibrahim A, Hassanien AE (2017). Linear discriminant analysis: a detailed tutorial. AI Commun..

[49] Iatan, I. F. The Fisher’s linear discriminant. In *Advances in Intelligent and Soft Computing* Vol. 77, 345–352 (Springer Berlin Heidelberg, 2010).

