# Supplementary material for: Endogenous fluctuations in cortical state selectively enhance different modes of sensory processing in human temporal lobe
Source: Nat Commun. 2023 Sep 11;14:5591. doi: 10.1038/s41467-023-41406-3 (PMC10495466; doi:10.1038/s41467-023-41406-3)
Supplement: Supplementary file 1 — Supplementary Information [file 41467_2023_41406_MOESM1_ESM.pdf]

SUPPLEMENTARY MATERIAL

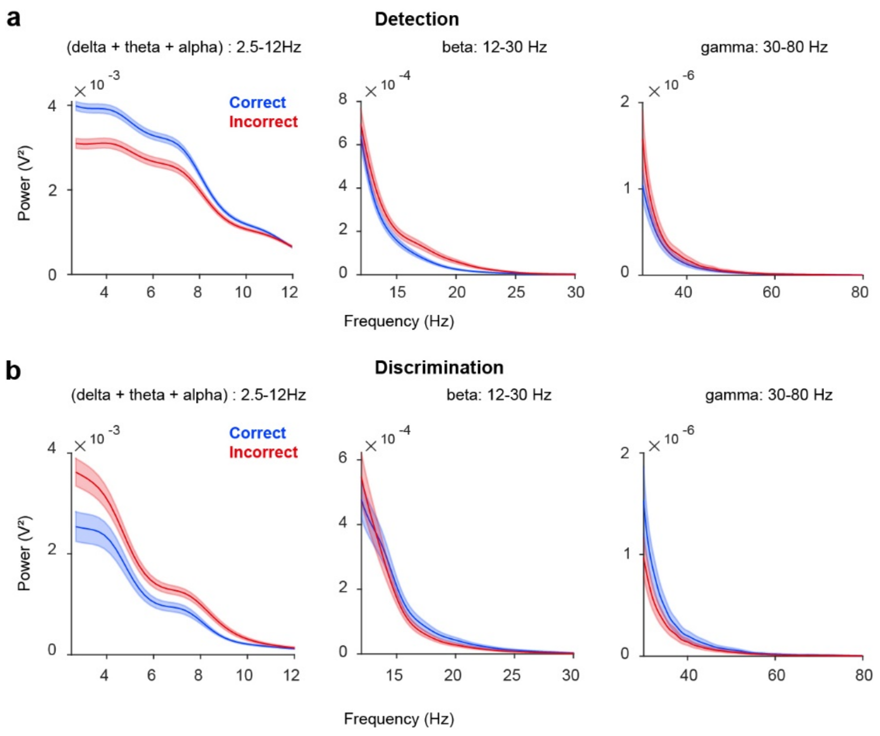

Figure S1: Power spectra for correct and incorrect trials. (a) Average power spectra of the IFP signal during the 400 ms pre-stimulus window depicting differences in average voltage-squared power between correct trials (blue) and incorrect trials (red) during detection task. **(b)** Same as (a) but for discrimination task. To improve the clarity of illustration, the total frequency band of interest (2.5-80 Hz) was divided into three distinct groups as follows: (delta + theta + alpha; 2.5-12 Hz; left), (beta; 12-30 Hz; middle), and (gamma; 30-80 Hz; right). Shaded regions represent standard error of mean.

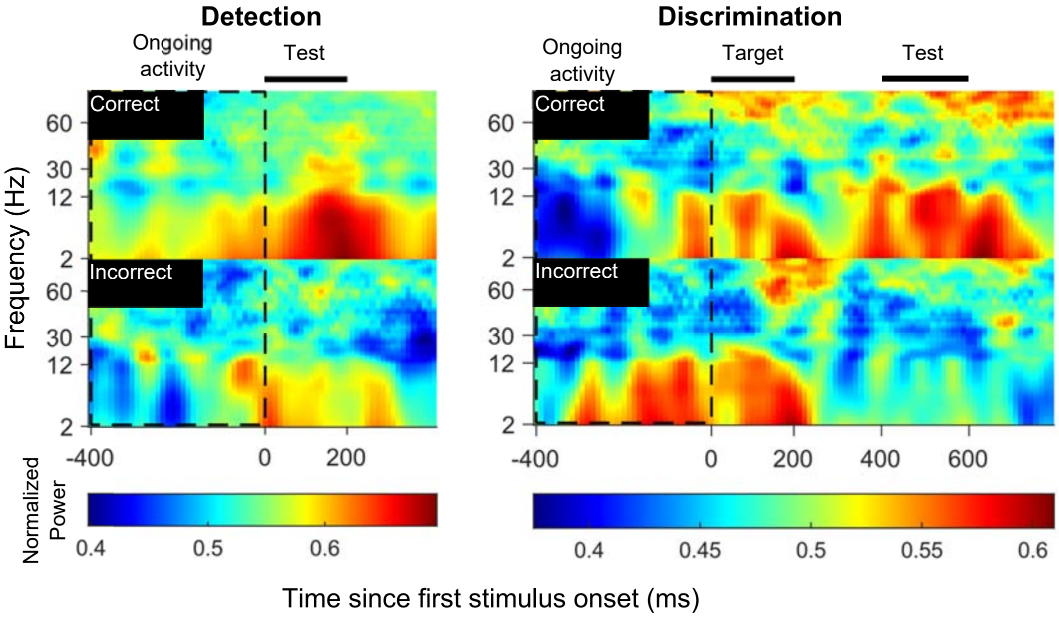

Figure S2. Average spectrograms: Average (n=97) spectrograms for both detection and discrimination tasks, highlighting the distinctions in ongoing IFP activity between correct and incorrect groups of trials. The magnitude values have been normalized between 0 and 1 for each frequency, and spectrograms from all electrodes across the four subjects have been pooled together.

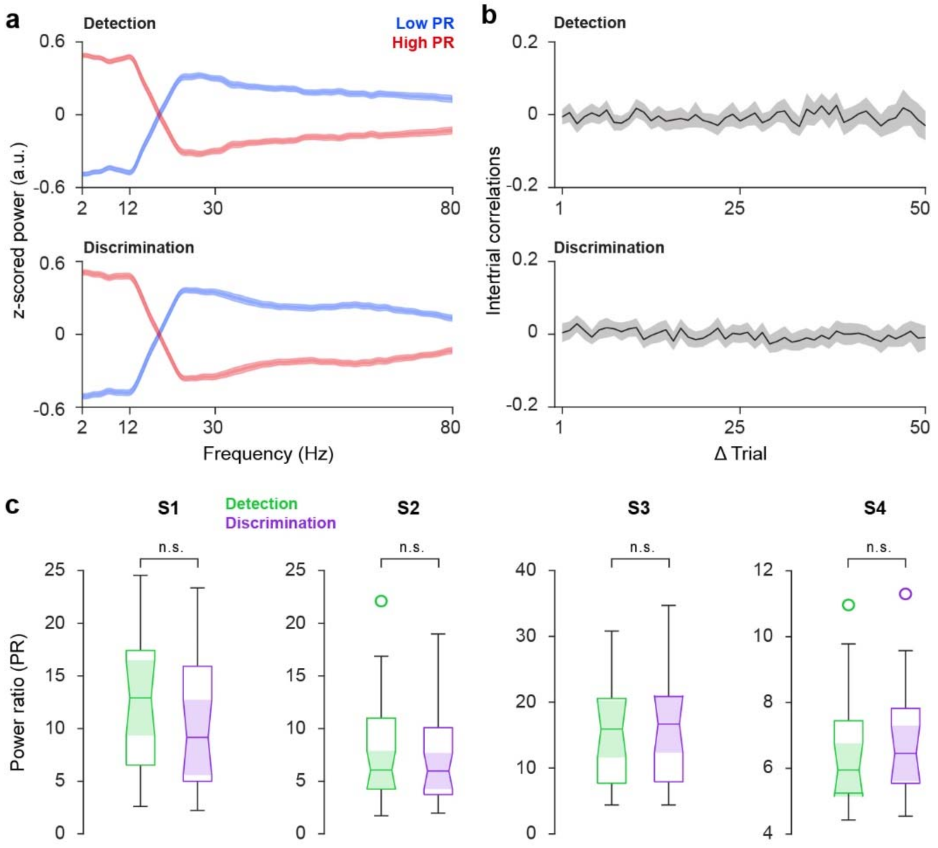

Figure S3. Power ratio (PR) during detection and discrimination tasks. (a) Average z-scored spectrum of the ECoG signal during the 400-ms pre-stimulus period for the low PR (blue) and high PR (red) groups of trials, pooling all the electrodes for both detection and discrimination tasks. Trials were divided into low or high PR groups based on whether the PR value in a trial was below or above the median PR value of all the trials. Shaded areas represent the standard error of means. **(b)** Average autocorrelation of pre-stimulus ongoing activity PR across trials for all the electrodes and sessions during both detection and discrimination tasks. Shaded areas represent the standard error of means. **(c)** Box plots comparing PR values during detection (green) and discrimination (purple) tasks across the four subjects. (ns: $p>0.05$, $n=97$ independent electrodes, two-sided Wilcoxon signed-rank test). The box plot's midline represents the median, the edges mark the quartiles, and the whiskers show the range. Notches indicate the 95% confidence interval of the median.

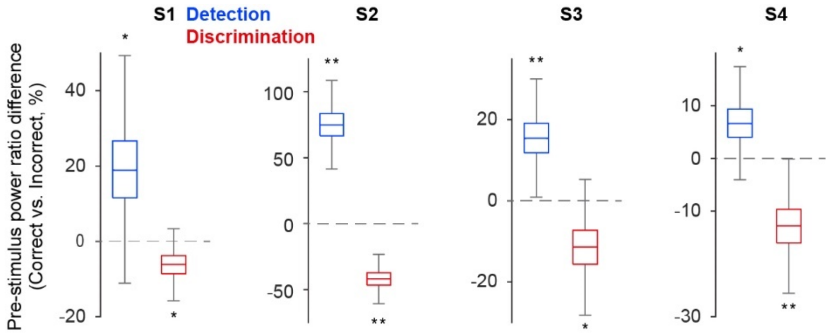

Figure S4: PR difference between correct and incorrect trials. Average percentage difference of ongoing PR between correct and incorrect trials for the detection (blue) and discrimination (red) tasks for the four subjects. PR values were significantly higher during correct trials compared to incorrect trials for the detection task across all four subjects (S1: +19.40%, p=0.032, n=23; S2: +75.29%, p<1e-6, n=34; S3: +15.537%, p=9e-4, n=22; S4: +6.755%, p=0.038, n=18; bootstrapping for positive mean with 1,000,000 iterations). Conversely, for the discrimination task, PR values were significantly lower during correct trials compared to incorrect trials for all four subjects (S1: -6.29%, p=0.028, n=23; S2: -42.06%, p<1e-6, n=34; S3: -11.56%, p=0.024, n=22; S4: -12.9%, p=0.001, n=18; bootstrapping for negative mean with 1,000,000 iterations), indicating highly robust effects: * p<0.05, ** p<0.01. The box plot's midline represents the median, the edges mark the quartiles, and the whiskers show the range. Notches indicate the 95% confidence interval of the median.

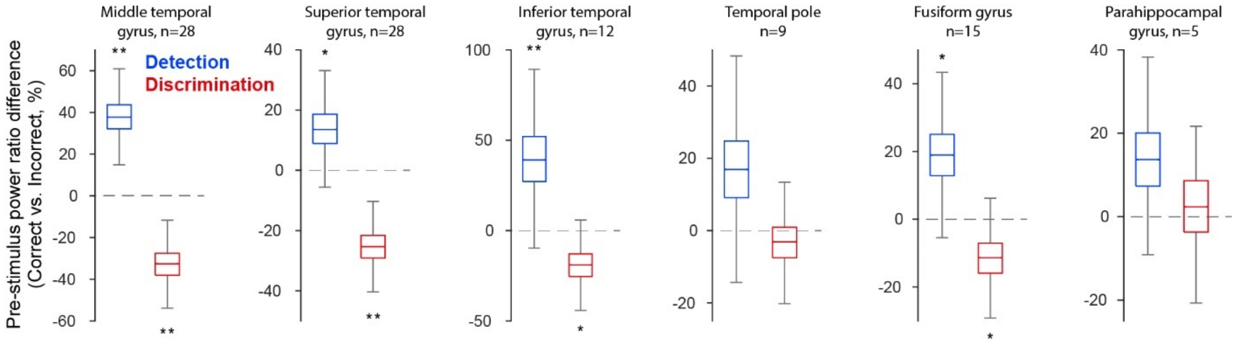

Figure S5. Differences in pre-stimulus ongoing power ratio. Population average of the percentage difference of ongoing PR between correct and incorrect trials for detection (Middle temporal gyrus: $p<1e-5$, Superior temporal gyrus: $p=0.012$, Inferior temporal gyrus: $p=0.002$, Temporal pole: $p=0.067$, Fusiform gyrus: $p=0.014$, Para-hippocampal gyrus: $p=0.06$; Bootstrapping for positive mean with 1e5 iterations) and discrimination (Middle temporal gyrus: $p<1e-5$, Superior temporal gyrus: $p<1e-5$, Inferior temporal gyrus: $p=0.011$, Temporal pole: $p=0.31$, Fusiform gyrus: $p=0.027$, Para-hippocampal gyrus: $p=0.63$; bootstrapping for negative mean with 100,000 iterations) tasks in six sub-regions of the temporal lobe, obtained by pooling together all the electrodes from the four subjects. *$p<0.05$, **$p<0.01$. The box plot's midline represents the median, the edges mark the quartiles, and the whiskers show the range. Notches indicate the 95% confidence interval of the median.

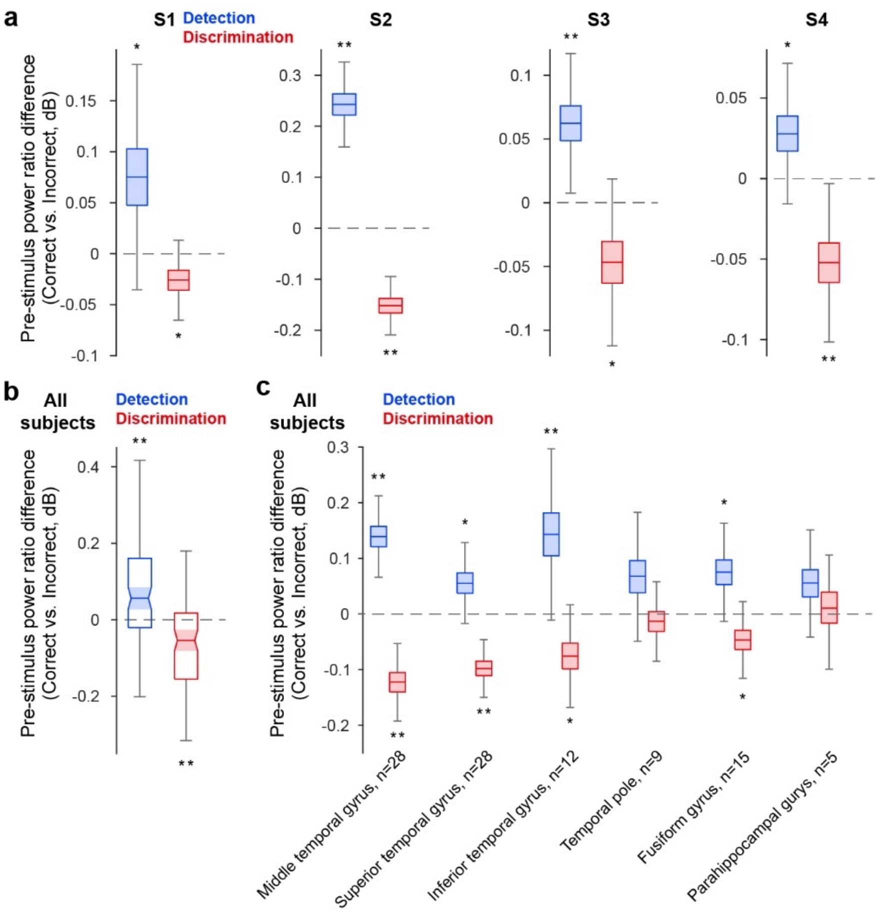

Figure S6: PR difference for correct and incorrect trials. (a) Average difference between ongoing activity PR in correct and incorrect trials for detection (blue) and discrimination (red) tasks for the four subjects. PR values are significantly higher during correct trials compared to incorrect for the detection task across all subjects (S1: p=0.032, n=23; S2: p<1e-6, n=34; S3: p=9e-4, n=22; S4: p=0.038, n=18; bootstrapping for positive mean with 1,000,000 iterations). For the discrimination task, PR values are significantly lower during correct trials compared to incorrect for all subjects (S1: p=0.029, n=23; S2: p<1e-6, n=34; S3: p=0.024, n=22; S4: p=0.001, n=18; bootstrapping for negative mean with 1,000,000 iterations) **(b)** Population average (n=97) of the difference between ongoing activity PR in correct and incorrect trials during detection (blue, p=1.35e-6, one-sided Wilcoxon signed-rank test for positive median) and discrimination (red, p=2.45e-7, one-sided Wilcoxon signed-rank test for negative median) tasks obtained by pooling all the electrodes across subjects. **(c)** Population average of the difference between ongoing activity PR in correct and incorrect trials for detection (blue; Middle temporal gyrus: p<1e-5, Superior temporal gyrus: p=0.012, Inferior temporal gyrus: p=0.002, Temporal pole: p=0.067, Fusiform gyrus: p=0.014, Para-hippocampal gyrus: p=0.06; bootstrapping for positive mean with 100,000 iterations)) and discrimination (red; Middle temporal gyrus: p<1e-5, Superior temporal gyrus: p<1e-5, Inferior temporal gyrus: p=0.011, Temporal pole: p=0.31, Fusiform gyrus: p=0.027, Para-hippocampal gyrus: p=0.63; bootstrapping for negative mean with 100,000 iterations) tasks in six sub-regions of the temporal lobe, obtained by pooling all the electrodes from the four subjects. *p<0.05, **p<0.01. The box plot's midline represents the median, edges mark the quartiles, and whiskers show the range. Notches indicate the 95% confidence interval of the median.

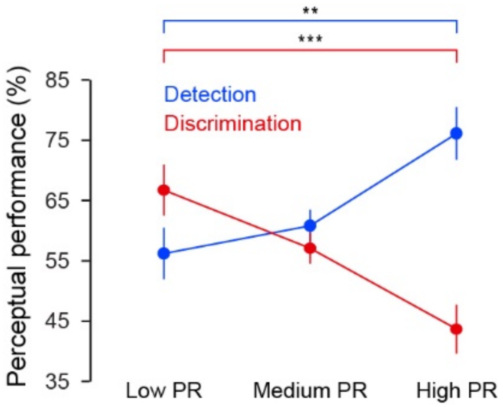

Figure S7: Perceptual performance as a function of PR. Perceptual performance for both detection (blue) and discrimination (red) tasks using three equally sized bins of PR values. The bins were created using PR percentiles: the low bin corresponds to values between 0-33.33, medium bin between 33.33-66.67, and high bin between 66.67-100 percentiles. Vertical lines represent standard error of means (** p=0.0023, *** p=0.0019, bootstrapping with 1,000,000 iterations).

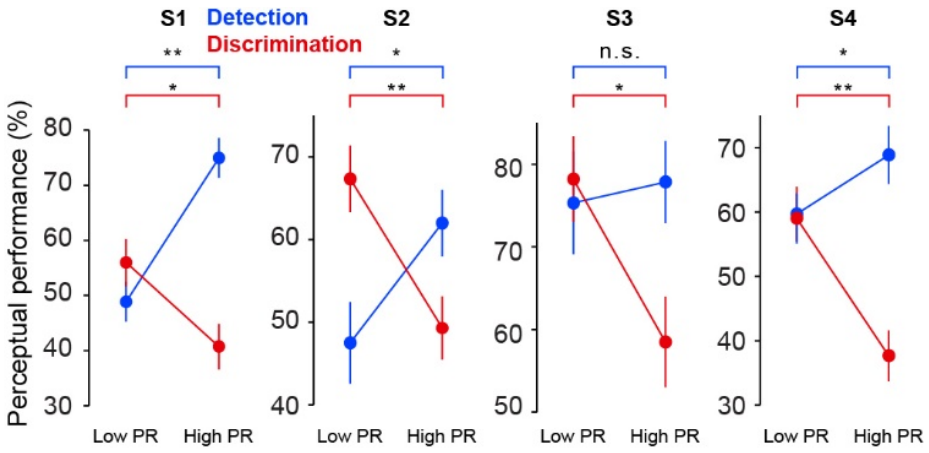

Figure S8: Effect of PR on perceptual performance. Perceptual performance in detection (blue) and discrimination (red) tasks across low and high ongoing PR state trials for the four subjects. Vertical bars represent standard error of mean. Trials with high PR values resulted in higher perceptual performance during the detection task (S1: 48.85±3.63 vs. 74.96±3.63, p=2e-6; S2: 47.5±4.92 vs. 62.01±4.02, p=0.029; S3: 75.32±6.2 vs. 77.85±4.95, p=0.35; S4: 59.71±4.28 vs. 68.88±4.51, p=0.04; mean±SEM; low vs. high PR trials; bootstrapping with 1,000,000 iterations), whereas trials with low PR values resulted in higher perceptual performance during the discrimination task (S1: 55.96±4.28 vs. 40.7±4.12, p=0.02; S2: 67.35±4.01 vs. 49.3±3.81, p=0.006; S3: 78.19±5.19 vs. 58.48±5.48, p=0.02; S4: 59.03±3.9 vs. 37.64±3.97, p=0.001; mean±SEM; low vs. high PR trials; bootstrapping with 1,000,000 iterations).

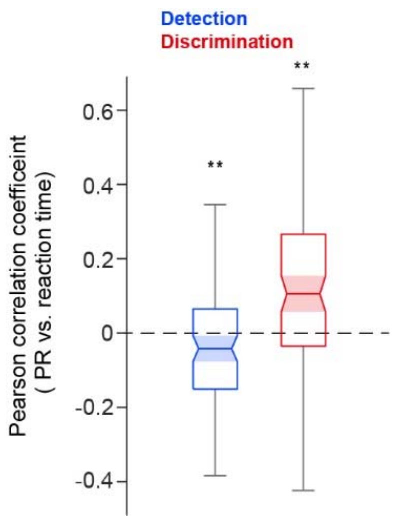

Figure S9: Relationship between pre-stimulus PR values and reaction times. Box plot summarizing the Pearson correlation coefficient between pre-stimulus PR values and the subsequent reaction times during correct trials in subjects performing detection (blue) and discrimination (red) tasks. We found a significant negative correlation between reaction time and PR values ($r=-0.042$, $p=0.006$, $n=97$ independent electrodes, one-sided Wilcoxon signed-rank test for negative median) in the detection task. In contrast, we found a significant positive correlation between reaction time and PR values ($r=0.11$, $p=2.4e-5$, $n=97$ independent electrodes, one-sided Wilcoxon signed-rank test for positive median) in the discrimination task. ** $p<0.01$. The box plot's midline represents the median, the edges mark the quartiles, and the whiskers show the range. Notches indicate the 95% confidence interval of the median.

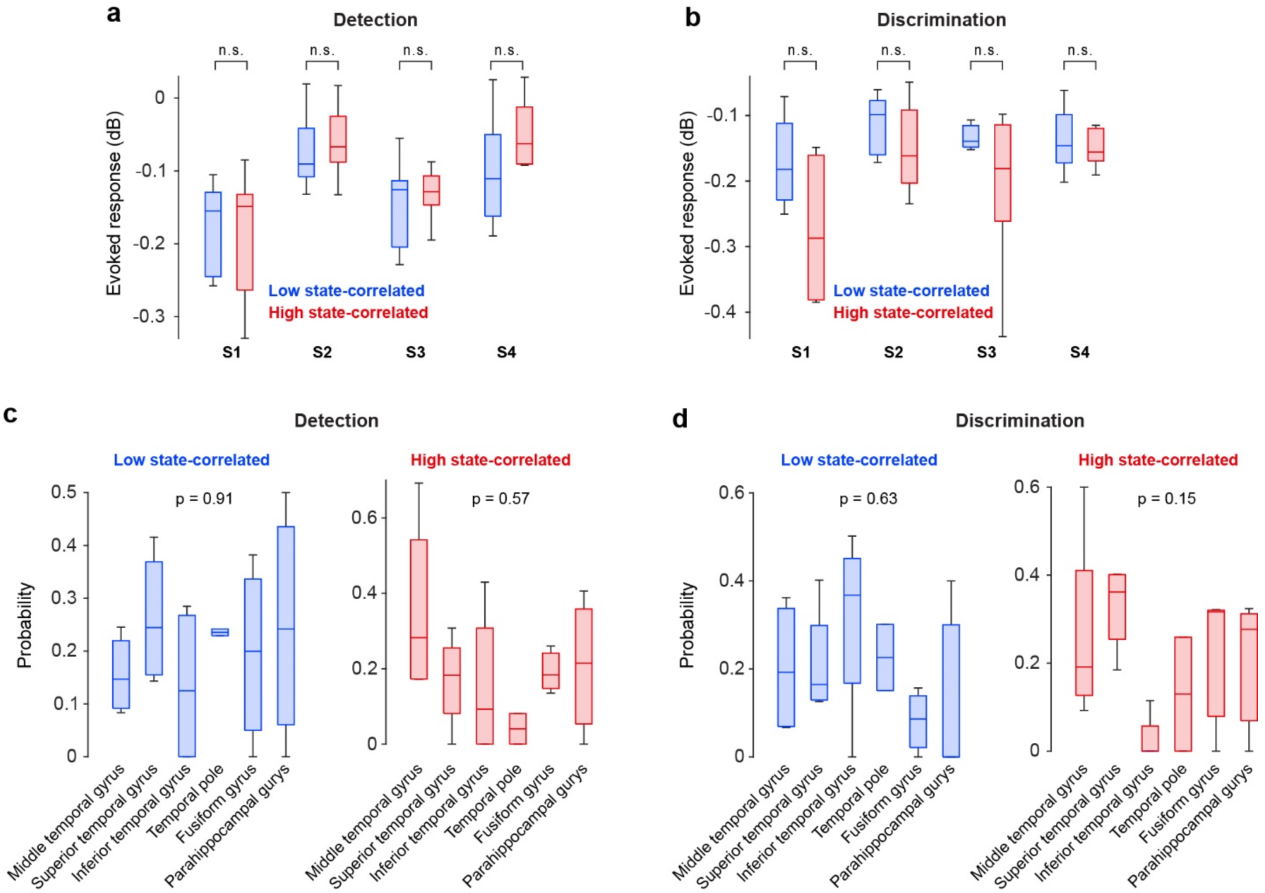

Figure S10: Comparison of evoked responses and electrode localization to sub-regions of the temporal lobe between low and high state-correlated electrodes. (a) Box plot comparing the evoked responses of low (blue) and high (red) state-correlated electrodes in the four subjects during the 200 ms test stimulus of the detection task (S1: n=8, S2: n=12, S3: n=7, S4: n=6). n.s. $p>0.05$, two-sided Wilcoxon rank sum test. **(b)** Same as (a) but for discrimination task. **(c)** Box plot illustrating the likelihood of an electrode, exhibiting low (blue) or high (red) state-correlation with the population, being positioned in various sub-regions of the temporal lobe in detection task. 'p' values are computed using Kruskal-Wallis multiple comparison test by ranks. (Middle temporal gyrus: n=4, Superior temporal gyrus: n=4, Inferior temporal gyrus: n=4, Temporal pole: n=2, Fusiform gyrus: n=3, Parahippocampal gyrus: n=3 independent subjects) **(d)** Same as (c) but for discrimination task. The box plot's midline represents the median, the edges mark the quartiles, and the whiskers show the range.

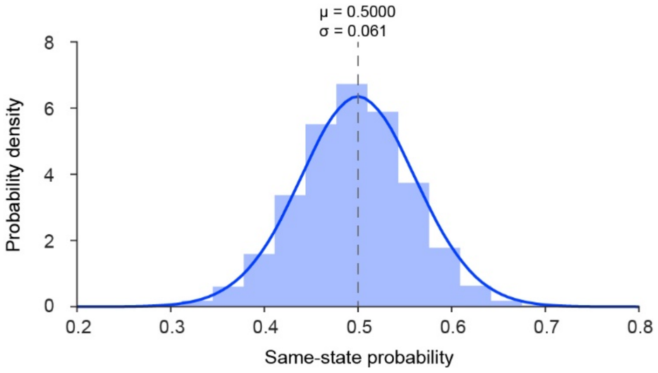

Figure S11: Chance level same-state probability. Distribution of same-state probabilities calculated after shuffling PR values at each individual electrode across trials. The mean of this distribution provided the chance level same-state probability: 0.5000 ± 0.061 (mean ± standard deviation).

		**Difference in pre-stimulus ongoing power ratio**					
		Detection			Discrimination		
Sub-region	Electrode Count	*Difference (%)*	*SEM*	*p-value*	*Difference (%)*	*SEM*	*p-value*
Middle temporal gyrus	*28*	38.02	8.52	<1e-5	-33.03	7.92	<1e-5
Superior temporal gyrus	*28*	14.03	7.16	0.012	-25.36	5.55	<1e-5
Inferior temporal gyrus	*12*	40.09	17.97	0.002	-19.39	9.28	0.011
Temporal Pole	*9*	16.97	11.31	0.067	-3.53	6.2	0.31
Fusiform gyrus	*15*	19.05	8.94	0.014	-11.64	6.49	0.027
Para-hippocampal gyrus	*5*	13.65	9.41	0.06	2.33	8.29	0.63

Supplementary Table T1. Population average of the percentage difference between correct and incorrect trial ongoing PR for detection and discrimination tasks in six sub-regions of the temporal lobe, obtained by pooling together all the electrodes from the whole population. p-values are obtained using bootstrapping with 100,000 iterations.
