# Supplementary material for: Endogenous fluctuations in cortical state selectively enhance different modes of sensory processing in human temporal lobe
Source: Nat Commun. 2023 Sep 11;14:5591. doi: 10.1038/s41467-023-41406-3 (PMC10495466; doi:10.1038/s41467-023-41406-3)
Supplement: Supplementary file 2 — Reporting Summary [file 41467_2023_41406_MOESM2_ESM.pdf]

nature portfolio

Corresponding author(s): Valentin Dragoi

Last updated by author(s): Aug 12, 2023

Reporting Summary

Nature Portfolio wishes to improve the reproducibility of the work that we publish. This form provides structure for consistency and transparency in reporting. For further information on Nature Portfolio policies, see our Editorial Policies and the Editorial Policy Checklist.

Statistics

For all statistical analyses, confirm that the following items are present in the figure legend, table legend, main text, or Methods section.

n/a	Confirmed	
☐	☒	The exact sample size (*n*) for each experimental group/condition, given as a discrete number and unit of measurement
☒	☐	A statement on whether measurements were taken from distinct samples or whether the same sample was measured repeatedly
☐	☒	The statistical test(s) used AND whether they are one- or two-sided<br>*Only common tests should be described solely by name; describe more complex techniques in the Methods section.*
☒	☐	A description of all covariates tested
☐	☒	A description of any assumptions or corrections, such as tests of normality and adjustment for multiple comparisons
☐	☒	A full description of the statistical parameters including central tendency (e.g. means) or other basic estimates (e.g. regression coefficient) AND variation (e.g. standard deviation) or associated estimates of uncertainty (e.g. confidence intervals)
☐	☒	For null hypothesis testing, the test statistic (e.g. *F*, *t*, *r*) with confidence intervals, effect sizes, degrees of freedom and *P* value noted<br>*Give P values as exact values whenever suitable.*
☒	☐	For Bayesian analysis, information on the choice of priors and Markov chain Monte Carlo settings
☒	☐	For hierarchical and complex designs, identification of the appropriate level for tests and full reporting of outcomes
☐	☒	Estimates of effect sizes (e.g. Cohen's *d*, Pearson's *r*), indicating how they were calculated

Our web collection on statistics for biologists contains articles on many of the points above.

Software and code

Policy information about availability of computer code

Data collection	Electrocorticogram (ECoG) signals were recorded using Nihon Kohden NeuroFax (Japan) with a recording bandwidth of 0.15 to 300 Hz.
Data analysis	Data analysis was performed using custom scripts written in MATLAB R2021b(Mathworks), Chronux 2.12 toolbox for MATLAB (chronux.org) and SciPy 1.11 (scipy.org) Python library using readily available functions.

For manuscripts utilizing custom algorithms or software that are central to the research but not yet described in published literature, software must be made available to editors and reviewers. We strongly encourage code deposition in a community repository (e.g. GitHub). See the Nature Portfolio guidelines for submitting code & software for further information.

Data

Policy information about availability of data

All manuscripts must include a data availability statement. This statement should provide the following information, where applicable:

- Accession codes, unique identifiers, or web links for publicly available datasets
- A description of any restrictions on data availability
- For clinical datasets or third party data, please ensure that the statement adheres to our policy

Source data used to generate the main and supplementary figures are provided as a Source Data file and can be accessed here https://zenodo.org/record/8241829. The raw datasets generated as part of this research are not publicly available due to containing information that does not comply with HIPAA regulations, and the human participants from whom the data were collected have not consented to their public release. However, they are available from the corresponding author upon request. Source data are provided with this paper.

Research involving human participants, their data, or biological material

Policy information about studies with human participants or human data. See also policy information about sex, gender (identity/presentation), and sexual orientation and race, ethnicity and racism.

Reporting on sex and gender	The research should not be dependent on the gender of the subjects and should apply equally to both males and females. Gender: 3 Males and 1 Female.
Reporting on race, ethnicity, or other socially relevant groupings	The results of the research should not be influenced by the race, ethnicity, or any other socially relevant characteristics of the participants.
Population characteristics	The findings of the research should be equally applicable to all age groups and genders. Population characteristics: (3 males, 1 female; 37.75 ± 7.95 years old, mean±SEM; 2 right-handed, 2 left-handed; 3 right implant, 1 left implant)
Recruitment	Subjects of the experiment were patients admitted into Memorial Hermann Hospital, Houston with subdural electrodes implanted on their brain's cortical surface to localize the foci of seizure and who had given informed consent about the experiments.
Ethics oversight	The University of Texas, Medical School at Houston committee for the protection of human subjects

Note that full information on the approval of the study protocol must also be provided in the manuscript.

Field-specific reporting

Please select the one below that is the best fit for your research. If you are not sure, read the appropriate sections before making your selection.

☒ Life sciences ☐ Behavioural & social sciences ☐ Ecological, evolutionary & environmental sciences

For a reference copy of the document with all sections, see nature.com/documents/nr-reporting-summary-flat.pdf

Life sciences study design

All studies must disclose on these points even when the disclosure is negative.

Sample size	Sample sizes are the number of ECoG electrodes (97) and the number of individual subjects (4). The effect was found to be consistent across subjects.
Data exclusions	Trials / electrodes contaminated by inter-icatal epileptic spikes were excluded from the analysis.
Replication	The findings were replicated across all 4 subjects.
Randomization	Samples (subjects/electrodes) were not assigned to multiple groups hence randomization of samples does not apply. However, different types of stimuli were randomly interleaved withing sessions.
Blinding	Blinding was not relevant as this study did not involve any clinical testing component.

Reporting for specific materials, systems and methods

We require information from authors about some types of materials, experimental systems and methods used in many studies. Here, indicate whether each material, system or method listed is relevant to your study. If you are not sure if a list item applies to your research, read the appropriate section before selecting a response.

Materials & experimental systems

n/a	Involved in the study
☒	☐ Antibodies
☒	☐ Eukaryotic cell lines
☒	☐ Palaeontology and archaeology
☒	☐ Animals and other organisms
☒	☐ Clinical data
☒	☐ Dual use research of concern
☒	☐ Plants

Methods

n/a	Involved in the study
☒	☐ ChIP-seq
☒	☐ Flow cytometry
☒	☐ MRI-based neuroimaging
